# Polygenic risk and hazard scores for Alzheimer's disease prediction

**DOI:** 10.1002/acn3.716

**Published:** 2019-02-18

**Authors:** Ganna Leonenko, Rebecca Sims, Maryam Shoai, Aura Frizzati, Paola Bossù, Gianfranco Spalletta, Nick C. Fox, Julie Williams, the GERAD consortium, John Hardy, Valentina Escott‐Price

**Affiliations:** ^1^ MRC Centre for Neuropsychiatric Genetics and Genomics Cardiff University Cardiff United Kingdom; ^2^ Institute of Neurology University College London London United Kingdom; ^3^ Department of Clinical and Behavioral Neurology Experimental Neuropsychobiology Laboratory IRCCS Santa Lucia Foundation Rome Italy; ^4^ UK Dementia Research Institute Cardiff University Cardiff United Kingdom

## Abstract

**Objective:**

Genome‐wide association studies (GWAS) have identified over 30 susceptibility loci associated with Alzheimer's disease (AD). Using AD GWAS data from the International Genomics of Alzheimer's Project (IGAP), Polygenic Risk Score (PRS) was successfully applied to predict life time risk of AD development. A recently introduced Polygenic Hazard Score (PHS) is able to quantify individuals with age‐specific genetic risk for AD. The aim of this study was to quantify the age‐specific genetic risk for AD with PRS and compare the results generated by PRS with those from PHS.

**Methods:**

Quantification of individual differences in age‐specific genetic risk for AD identified by the PRS, was performed with Cox Regression on 9903 (2626 cases and 7277 controls) individuals from the Genetic and Environmental Risk in Alzheimer's Disease consortium (GERAD). Polygenic Hazard Scores were generated for the same individuals. The age‐specific genetic risk for AD identified by the PRS was compared with that generated by the PHS. This was repeated using varying SNPs *P*‐value thresholds for disease association.

**Results:**

Polygenic Risk Score significantly predicted the risk associated with age at AD onset when SNPs were preselected for association to AD at *P* ≤ 0.001. The strongest effect (*B* = 0.28, SE = 0.04, *P* = 2.5 × 10^−12^) was observed for PRS based upon genome‐wide significant SNPs (*P* ≤ 5 × 10^−8^). The strength of association was weaker with less stringent SNP selection thresholds.

**Interpretation:**

Both PRS and PHS can be used to predict an age‐specific risk for developing AD. The PHS approach uses SNP effect sizes derived with the Cox Proportional Hazard Regression model. When SNPs were selected based upon AD GWAS case/control *P* ≤ 10^−3^, we found no advantage of using SNP effects sizes calculated with the Cox Proportional Hazard Regression model in our study. When SNPs are selected for association with AD risk at *P *> 10^−3^, the age‐specific risk prediction results are not significant for either PRS or PHS. However PHS could be more advantageous than PRS of age specific AD risk predictions when SNPs are prioritized for association with AD age at onset (i.e., powerful Cox Regression GWAS study).

## Introduction

Alzheimer's disease (AD) is the most common form of neurodegenerative disorder^1^ with over 47 million people affected worldwide and a global economic impact estimated at about US $818 billion.^2^

AD is highly heritable with an estimated 80% of the liability explained by genetic factors.^3^ Risk for developing AD involves multiple genetic and environmental components, with *APOE* genotype^4^ having the strongest genetic effect.^5^ In the last 20 years numerous relevant susceptibility loci, genes, and pathways have been identified that will improve understanding of this complex disease and identify potential therapeutic targets. The largest Genome‐Wide association study (GWAS) identified more than 20 loci^6^ associated with late (after the age of 65 years) onset AD (LOAD). The analysis was extended to biological pathways with enrichment in immune response, regulation of endocytosis, cholesterol response, and proteasome‐ubiquitin activity pathways.^7^

The development and validation of AD prediction algorithms is a very important step towards therapeutic strategies for AD prevention and intervention. A polygenic risk score (PRS) approach has demonstrated 75–84% prediction accuracy of AD risk with *APOE*, the polygenic score, sex and age as predictors.^8^, ^9^ PRS is constructed as a weighted sum of allele counts, where the weights are the B‐coefficients of SNP association with the disease obtained with the Logistic Regression (LR) analysis. Recent development of a polygenic hazard score (PHS) approach goes beyond AD risk prediction and provides prediction of individual age‐specific risk for developing AD.^22^ Prior to PHS analyses, Desikan et al.^10^ selected SNPs based on their association with AD at *P* ≤ 10^−5^ in the publically available IGAP dataset. Then they constructed PHS in a similar way to PRS, with the exception that PHS uses log(HR) as SNP risk allele weighs, instead of log(OR). Both, log(HR)s and the best PHS model were identified by running step‐wise Cox regression, yielding 31 SNPs in addition to *APOE ε*2 and *ε*4 alleles (see Desikan et al.^10^).

LR and Cox Proportional Hazard Regression (Cox regression) analyses are widely used in epidemiological studies depending upon the question of interest and available information. LR is used to measure the relationship between a binary variable (e.g., case/control) and predictor variables, while Cox regression investigates the association between the time‐to‐event (“survival time”) of patients together with other predictor variables. Cox regression^11^ is one of the most‐widely applied methods in medical studies when investigating time‐dependent explanatory variable. Similar to odds ratio (OR) in LR, Cox regression estimates the hazard ratio (HR) that is a measure between the probability of events in a “case” group compared to the probability of events in a “control” group. The advantage of Cox regression over LR is that the former estimates an instantaneous risk for developing AD, based on genotype and age, while the latter ignores “survival time” and censoring information.

There have been several studies conducted to compare Logistic and Cox regression models.^12^, ^13^, ^14^, ^15^ Earlier studies have shown that if the time‐to‐event data are available, Cox proportional hazards models have more statistical power to detect risk factors than LR models,^16^ since it accounts for the time until events occur. However, the two models yield similar estimates of regression coefficients in studies with short follow‐up (5 years or less) time and high survival rate. In addition, although these two regression models have different purposes, it has been shown that the risk factors with strong effect size will be significant in both models and present similar regression coefficient estimates.^14^, ^17^

The aim of this study was to quantify the age‐specific genetic risk for AD with PRS and compare the results with PHS. Previously, 31 SNPs in addition to *APOE ε*2 and *ε*4 alleles were identified^10^ that were used in deriving PHS and then predicting the age‐specific individual risk. There are two components in the PHS/PRS analyses, (1) how the individual score is generated, that is, how the SNPs were selected and their effect sizes are derived, and (2) what aspect of the disease is predicted by this score (age at onset or overall risk).

First, we compared the effect sizes of SNP association to AD derived in the same dataset using two different models (1) Logistic regression and (2) Cox regression. To explore the second component, we compared the accuracy in quantification of individual differences in age‐specific genetic risk for AD using PRS, that is, when the SNP effect sizes are derived by LR, and PHS when the effect sizes are derived by Cox regression.

Tan et al.^18^ in their editorial claim that PHS has advantage over PRS to help inform disease management decisions for at‐risk individuals in the clinic. Our study shows that PHS, as constructed in Desikan et al.^10^ gives very similar results to PRS when tested in the Genetic and Environmental Risk for Alzheimer's disease (GERAD) dataset.

## Materials and Methods

### Data

Generation of PRS (and similar PHS) requires two independent datasets: a discovery sample, where the summary statistics are sufficient, and a validation samples, which is independent of the discovery sample and contains genotypes for each individual.^19^ We compared individual SNP hazard ratio (HR)^10^ with odds ratios (OR) reported by the International genomics of Alzheimer's project (IGAP) GWAS.^6^ The original IGAP summary statistics was derived using a meta‐analysis of four GWAS datasets, namely European Alzheimer ‘s disease Initiative (EADI), Cohorts for Heart and Aging Research in Genomic Epidemiology (CHARGE), Alzheimer's Disease Genetics Consortium (ADGC) and Genetic and Environmental Risk for Alzheimer's disease (GERAD) consortia.

In this study, since individual genotypes of the GERAD sample were available to us, we used GERAD data as the test set. For PRS calculation we used SNP's log(OR) from the meta‐analysis of three consortia (EADI, CHARGE and ADGC), excluding GERAD, hereby referred to as IGAP_noGERAD. Note that individual SNP effects (*B* = log(OR)) of IGAP_noGEARD dataset were adjusted for age. To generate polygenic hazard scores in the GERAD sample, we used hazards ratio estimates for the 31 SNPs reported in ADGC data.^10^ To test other SNPs, we split the GERAD data and estimated hazards ratios in 75% of the data and generated and tested individual scores in the remaining 25% of the data.

The GERAD dataset consists of 3177 cases and 7277 controls of Caucasian ancestry (see Table S1 for cohort statistical characteristics) and partly was published previously.^20^ Haplotype Reference Consortium (HRC), version r1.1 2016, was used to impute GERAD genotype data on the Michigan Imputation Server,^21^ which to date, allows the most accurate imputation of genetic variants. Imputed genotype probabilities (also known as dosages) were converted to most probable genotype with a probability threshold of 0.9 and greater. SNPs were removed if their imputation INFO‐score < 0.4, MAF < 0.1, missingness of genotypes ≥ 0.05 or HWE < 10^−6^. A total of 6,119,694 variants were retained. To correct for population structure and genotyping differences, all our analyses were adjusted for gender and 3 principle components.^20^

For the survival analysis model we used age at onset where available, and imputed age at onset for the remaining individuals. Imputed age at onset was estimated by subtracting 5 years from the age of the last assessment (as the mean difference between the age of the last assessment and age at onset was 4.7 years in our data). The age at onset has been imputed for 253 (8%) cases and for an additional 551 individuals had no age related information. Out of the remaining 9903 individuals (2626 cases and 7277 controls), *APOE* genotypes were available for 8415 individuals (2384 cases and 6031 controls) and these subjects were included in the analysis.

To validate the PHS approach in a sample which is independent of ADGC,^10^ we split our GERAD data into a discovery (75% or 1934 cases and 5493 controls) dataset, for estimation the HR, and validation (25% or 692 cases and 1784 controls) dataset, where we derived the PHS for each individual and tested for age‐specific risk prediction.

**Figure 1:**
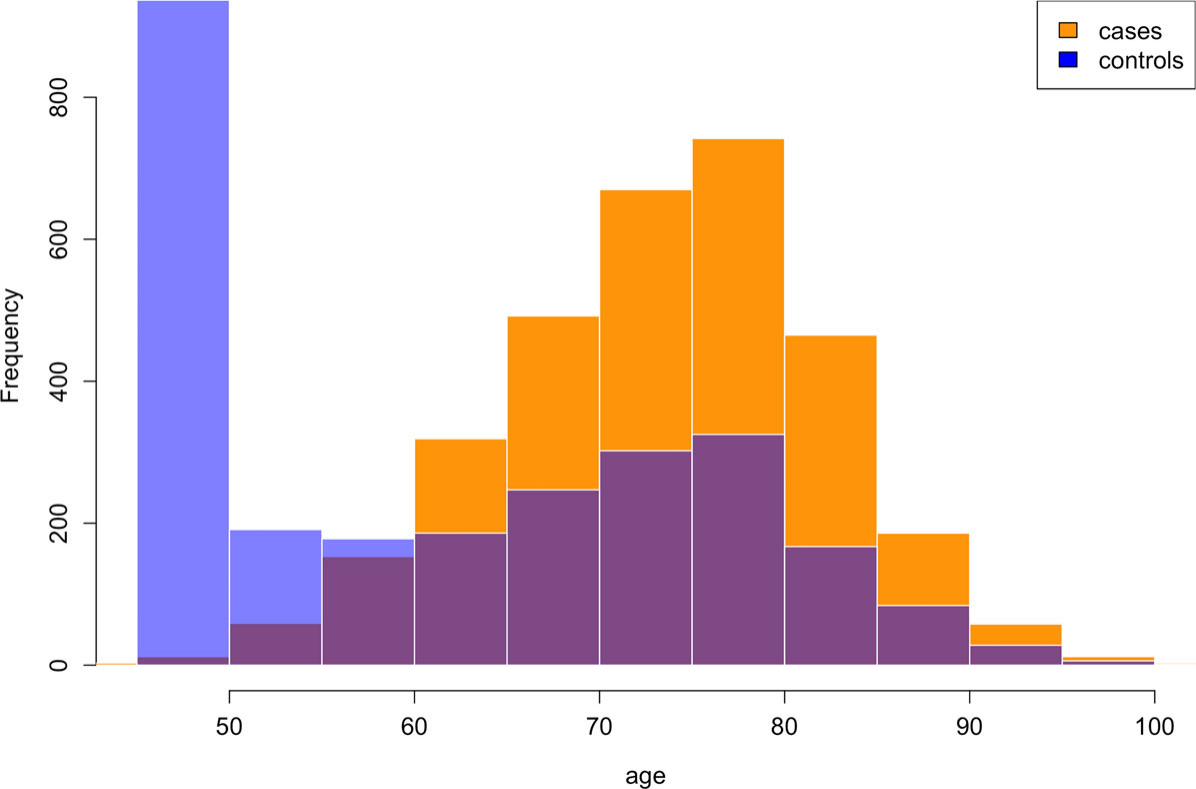
Histogram of age of AD cases and controls in the GERAD dataset.

In the GERAD sample 5570 controls were from the 1958 birth cohort (all included at age 45) (http://www.b58cgene.sgul.ac.uk), introducing differences in age distribution between cases and controls (Fig. 1). To avoid a potential bias due to these differences, we have repeated the analyses including only participants with age 55 and above, retaining 4100 individuals (2575 cases and 1525 controls).

### Statistical analysis

We calculated PHS for each subject in our GERAD sample using effect sizes as reported in the Desikan et al.^10^(see fifth column of Table 1). The PRS were generated using IGAP_noGERAD effect sizes for the 25 SNPs reported (see seventh column of Table 1). To evaluate the contribution of the 25 SNPs over and above *APOE ε*2 and *ε*4 risk alleles, PHS and PRS were derived in three ways (1) only using *ε*2 and *ε*4 risk alleles, (2) 25 SNPs, and (3) combining *ε*2, *ε*4, and 25 SNPs. We tested whether the addition of either PHS or PRS into the Cox regression model improves the model fit over and above *APOE ε*4 and *ε*2 risk alleles using anova() function in R. Since *APOE* is the strongest predictor of AD risk, we also validated our results in *ε*3 homozygous individuals (*N* = 4368). Furthermore, to investigate the stability of the results o for the 25 SNPs of interest, this analysis was repeated for randomly selected subsets of cases and controls (20, 40, 60, 80, and 100% of the whole sample).

**Table 1:** *APOE* variants and the 31 SNPs and, their closest genes, log hazard ratio estimates used for PHS construction in Desikan et al.^10^ and their odds ratio estimates as in Lambert et al.^6^

SNP	Chr	Position	Gene	Β = log(HR) Desikan et al. (2017)	−log10(*P*‐value) Desikan et al. (2017)	*B* = log(OR) in IGAP	*B* = log(OR) IGAP_ noGERAD	A1 IGAP_ noGERAD
*APOE ε*2	19		*APOE*	−0.47	>15.0	−0.66 1	−0.49^2^	*ε*2
*APOE ε*4	19		*APOE*	1.03	>20.0	1.12 2	0.66^2^	*ε*4
rs4266886	1	207685786	CR1	−0.09	2.7	−0.1542	0.1520	T
rs61822977	1	207796065	CR1	−0.08	2.8	−0.0805	−0.0820	A
rs6733839	2	127892810	BIN1	−0.15	10.5	−0.1880	0.1807	T
rs10202748	2	234003117	INPP5D	−0.06	2.1	−0.058	−0.0603	A
rs115124923	6	32510482	HLA‐DRB5	0.17	7.4	0.1216	−0.0973	A
rs115675626	6	32669833	HLA‐DQB1	−0.11	3.2	−0.1246	0.1040	A
rs1109581	6	47678182	GPR115	−0.07	2.6	−0.0651	0.0601	T
rs17265593	7	37619922	BC043356	−0.23	3.6	−0.0659	−0.0620	T
rs2597283	7	37690507	BC043356	0.28	4.7	0.0679	0.0629	A
rs1476679	7	100004446	ZCWPW1	0.11	4.9	0.1741	0.0712	T
rs78571833	7	143122924	AL833583	0.14	3.8	0.0795	0.2083	A
rs12679874	8	27230819	PTK2B	−0.09	4.2	−0.0795	−0.0748	A
rs2741342	8	27330096	CHRNA2	0.09	2.9	0.0916	−0.0872	T
rs7831810	8	27430506	CLU	0.09	3.0	0.083	−0.0774	A
rs1532277	8	27466181	CLU	0.21	8.3	0.1385	−0.1271	T
rs9331888	8	27468862	CLU	0.16	5.1	0.0819	−0.0806	C
rs7920721	10	11720308	CR595071	−0.07	2.9	−0.0713	−0.0660	A
rs3740688	11	47380340	SPI1	0.07	2.8	0.0724	0.0739	T
rs7116190	11	59964992	MS4A6A	0.08	3.9	0.0991	−0.0968	A
rs526904	11	85811364	PICALM	−0.20	2.3	−0.1188	−0.1130	T
rs543293	11	85820077	PICALM	0.30	4.2	0.1257	−0.1192	A
rs11218343	11	121435587	SORL1	0.18	2.8	0.2697	0.2539	T
rs6572869	14	53353454	FERMT2	−0.11	3.0	−0.0947	0.1006	A
rs12590273	14	92934120	SLC24A4	0.10	3.5	0.1348	0.1231	T
rs7145100	14	107160690	abParts	0.08	2.0	0.1047	−0.1081	C
rs74615166	15	64725490	TRIP4	−0.23	3.1	−0.3358	−0.2986	T
rs2526378	17	56404349	BZRAP1	0.09	4.9	0.0762	0.0754	A
rs117481827	19	1021627	C19orf6	−0.09	2.5	−0.1288	−0.1059	T
rs7408475	19	1050130	ABCA7	0.18	4.3	0.0971	−0.0973	C
rs3752246	19	1056492	ABCA7	−0.25	8.4	−0.1345	−0.1308	C
rs7274581	20	55018260	CASS4	0.10	2.1	0.139	0.1497	A

First 6th columns are the same as they were presented in Desikan et al. (2017),^10^ followed by effect sizes from IGAP and IGAP_noGERAD summary statistics and reference allele as it was presented in IGAP.

1 *B* estimated on GERAD data when running LR.

2 *B* estimated on GERAD data when running Cox regression.

We further investigated whether a different (larger) set of SNPs could improve the power of the association and the quality of the prediction of age‐specific genetic risk for AD. For this we used the full GERAD GWAS dataset (4,997,262 SNPs). The dataset was LD pruned with *r* ^2 ^= 0.1 in a 1000 kb window, retaining 167,188 SNPs (with IGAP_noGERAD summary statistics). A fivefold cross‐validation approach was employed splitting the GERAD data randomly into 75%/25% discovery and validation sets, respectively. LR and Cox regression were run in the discovery (75% of GERAD) dataset in order to obtain effect sizes, log(OR) and log(HR), respectively. The PRS and PHS for each individual in the validation set (25% of GERAD) were generated for AD association *P*‐value thresholds of *P* ≤ 5 × 10^−8^, 10^−5^, 10^−3^, 0.05, 0.1, and 0.5, adjusted for covariates and then standardized. The Cox regression model was run to predict age‐specific genetic risk for AD by (1) PHS and (2) PRS in the validation set. Note, in LR analysis age was not included into the model, while Cox regression model has accounted for the age as a censor variable. The proportional hazards assumption for the Cox regression model, that is, that the hazard rate ratio is constant over time, was tested using function cox.zph() in R. *P*‐values of the proportional hazards assumption tests were non‐significant indicating that the models were correctly specified.

The results of the cross‐validation procedure are reported as mean and SD of the effect sizes, and as the average of the *P*‐values across cross‐validation. We also report the average of the correlation coefficients between individual PHS vs PRS scores.

## Results

**Figure 2:**
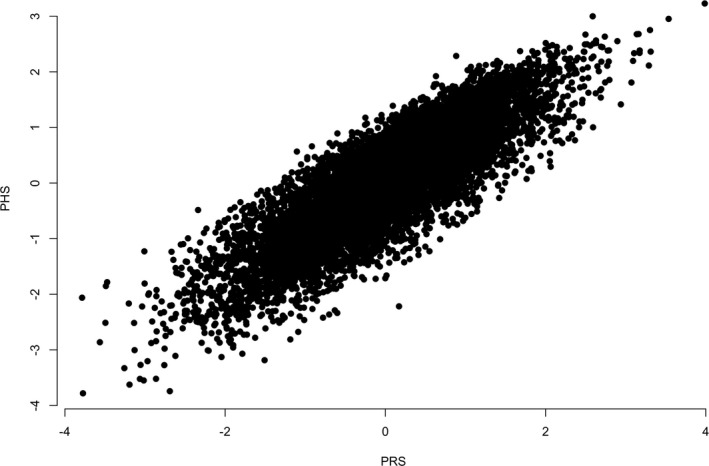
Scatter plot of individual's PRS and PHS that were derived using 25 SNPs from Desikan et al.^10^ in the GERAD sample.

In attempt to directly replicate the Desikan et al.^10^ results, we performed analysis on 8415 individuals (2384 cases and 6031 controls) for whom *APOE* genotypes were available. PHS was derived for each individual in the GERAD dataset with *APOE ε*2 and *ε*4 risk alleles and the 25 SNPs available in the dataset that were reported in Desikan et al.^10^ (see also Table 1). PRS was derived using effect sizes obtained from IGAP_noGERAD summary statistics using the same SNPs as for PHS. As the effect sizes for *APOE ε*2 and *ε*4 risk alleles in IGAP_noGERAD data were unavailable, we used GERAD data to estimate them using LR (*B*(*ε*2) = −0.66, *B*(*ε*4) = 1.12). Table 2 presents the results of PHS and PRS models, where risk scores were constructed with (1) *APOE ε*4 and *ε*2 risk alleles (columns 2 and 3), (2) PHS and PRS scores based upon 25 SNPs (columns 4 and 5), and (3) PHS and PRS scores + *APOE ε*4 and *ε*2 risk alleles (columns 6 and 7). It can be seen from Table 2 that the regression coefficient estimates (*B*) and *P*‐values for both models are very similar. In both models the strongest association is observed when the risk scores (either PHR or PRS) were constructed using *APOE ε*2 and *ε*4 risk alleles. The scores derived with the 25 SNPs also show significant association with age‐specific AD risk (*P* = 9.7 × 10^−8^, *P* = 1.9 × 10^−10^ for PHS and PRS Models, respectively). The last column of Table 2 compares the fit of the *APOE*‐alone model with the model using *APOE *+ 25 SNPs as predictors and shows that the both PHS and PRS significantly improve the association results over and above the predictor variable based upon *APOE ε*2 and *ε*4 only. The correlation between individual PHS and PRS was *r* = 0.85 (see Fig. 2) and even higher when *APOE* variants were included *r* = 0.99. Analysis in *ε*3 homozygotes (*N* cases = 846, *N* controls = 3522) confirmed that PRS is a significant predictor of age specific risk for AD over and above *APOE* (*B* = 0.17, *P* = 9.5 × 10^−7^), and revealed that PHS is a slightly less significant predictor (*B* = 0.14, *P* = 2.0 × 10^−5^).

**Table 2:** Cox regression analysis results using PHS and PRS based upon (the same) SNPs as in Desikan et al. (2017)^10^ in GERAD dataset

	*APOE*(*ε*2 + *ε*4)		25 SNPs		*APOE*(*ε*2 + *ε*4) + 25 SNPs		Compare: APOE vs. 25 SNPs + *APOE*(*ε*2 + *ε*4) *P*‐value
	*B* [SE]	*P*‐value	*B* [SE]	*P*‐value	*B* 1 [SE], *B* 2 [SE]	*P*‐value	
PHS model with effect sizes from Desikan et al. (2017)	0.41 [0.019]	1.8 × 10^−101^	0.11 [0.02]	9.7 × 10^−8^	0.41 0.019], 0.11 [0.02]	4.8 × 10^−103^	3.4 × 10^−8^
PRS model with effects from IGAP_noGERAD	0.408 [0.018]	2.7 × 10^−103^	0.13 [0.02]	1.9 × 10^−10^	0.41 [0.019], 0.13 [0.02]	2.4 × 10^−105^	8.8 × 10^−10^

1 *B* is a coefficient for *APOE* (*ε*2 + *ε*4).

2 *B* is a coefficient for PRS/PHS without *APOE*.

**Figure 3:**
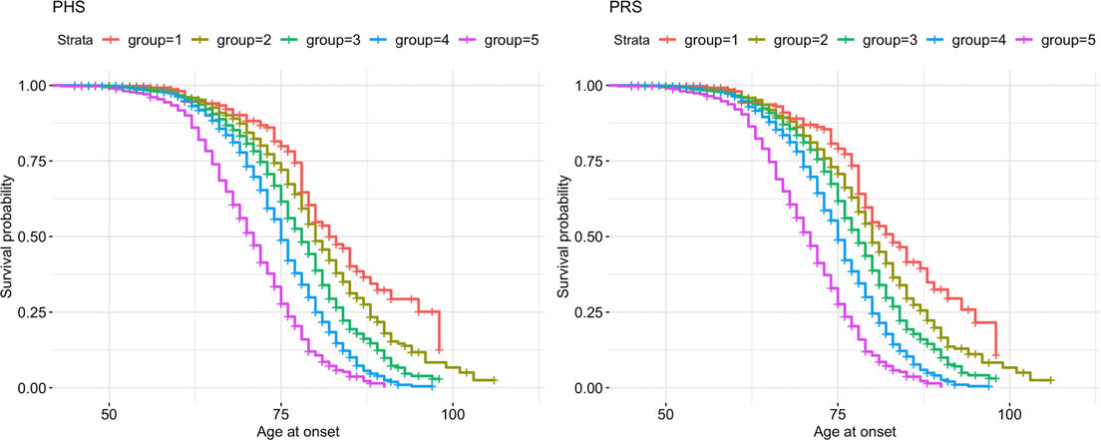
Survival curves for PHS and PRS scores + *APOE ε*4 and *ε*2 risk alleles for 8,415 individuals (2,384 cases and 6,031 controls) for whom *APOE* genotypes were available. Individuals are split into 5 groups based on 0–5%, 5–25%, 25–75%, 75–95%, and 95–100% of PHS/PRS distributions.

Figure 3 shows survival curves for individuals quantified in 5 groups based on 0–5%, 5–25%, 25–75%, 75–95%, and 95–100% of PHS/PRS distributions. Survival curves were created using PHS (left panel) and PRS (right panel) accounting for *APOE ε*2, *ε*4 risk alleles and the 25 SNPs. There is a clear difference in age at onset for individuals that belong to different distribution groups for both models. For example, individuals with the low PHS (bottom 5% of the PHS distribution, purple line in the left panel of Fig. 3) on average have 20 years earlier age at onset as compared to the top 5% of the PHS (red line in the left panel of Fig. 3), given a probability of 0.25 of developing AD. For both models the curves are almost identical and there is clear difference in age at onset for individuals that belong to different distribution groups for both models.

**Figure 4:**
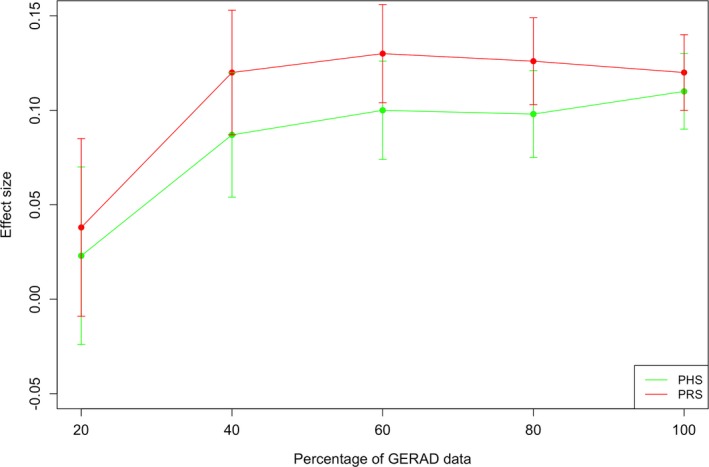
Results of age‐specific predictions using PRS and PHS analyses in GERAD subsamples of 20, 40, 60, 80, and 100% randomly selected individuals. The PHS and PRS are derived based upon 25 SNPs reported by Desikan et al.^10^

The results of age‐specific predictions using PHS and PRS analyses based upon 25 SNPs for different sample sizes are presented in Figure 4. As before, the age‐specific risk effect sizes for both PRS and PHS were similar and (as expected) were lowest in the smallest subset of the GERAD data, gradually increasing with sample size.

To increase the power of the analysis we attempted to increase the sample size and the number of SNPs included in the analysis. We utilized the full GERAD dataset (2626 cases and 7277 controls), and included *APOE* locus via the best (imputed) proxies as not all individuals in the GEARD sample had direct *APOE* genotypes. The correlation between *APOE ε*2 allele and rs41290120 was *r* = 0.74 and between *APOE ε*4 allele and rs7259620 was *r* = −0.41. To include more SNPs into the PHS, we increased the *P*‐value association threshold to 0.5 and LD pruned SNPs while keeping the most significantly AD‐associated SNPs (in IGAP_noGERAD) for the analysis. For the pruned SNPs we re‐estimated the *B* coefficients with Cox regression and LR in the 75% of the GERAD sample, and ran the age specific prediction analyses in the remaining 25% with fivefold cross‐validation (see Results in Table 3). The Cox regression analysis suggests that significant age‐specific prediction when using either PHS or PRS is achieved with the SNP selection threshold at *P *≤ 10^−3^. The Pearson's correlation coefficient between individual PHS and PRS is high for SNP selection thresholds up to *P *≤ 10^−5^ (Table 3, last column). Age‐specific predictions are no longer significant when SNPs *P*‐value thresholds are higher than 10^−3^. Since the age distribution was quite different in cases and controls (see Fig. 1), the same analyses were performed for individuals aged 55 and above, retaining 4100 samples for the analysis (see Results in Table 4). Despite the substantial reduction in sample size, the pattern of the results stays the same across tables.

**Table 3:** Cox regression analyses results of 5‐fold cross‐validation for PHS and PRS in GERAD dataset

SNP selection *P*‐value threshold	*N* SNPs	PHS		PRS		Correlation between PHS and PRS
		*B* [SD]	*P*‐value	*B* [SD]	*P*‐value	
5 × 10^−8^	31	0.28 [0.04]	4.3 × 10^−13^	0.28 [0.04]	2.5 × 10^−12^	0.96
10^−5^	80	0.26 [0.02]	2.8 × 10^−11^	0.29 [0.05]	5.7 × 10^−11^	0.79
10^−3^	1460	0.13 [0.025]	3.4 × 10^−3^	0.18 [0.04]	1.9 × 10^−4^	0.17
0.05	29998	0.07 [0.027]	0.12	0.1 [0.034]	0.04	0.21
0.1	49247	0.06 [0.031]	0.18	0.09 [0.028]	0.05	0.27
0.5	128952	0.08 [0.024]	0.07	0.08 [0.035]	0.13	0.42

First column shows the *P*‐value thresholds for AD associated SNP selection (from an independent IGAP_noGERAD data). Second column represents the number of SNPs that were included to the PRS/PHS score. PHS and PRS effect sizes (mean and SD) and averaged *P*‐values across 5‐fold cross‐validation subsampling are shown in columns 3–6. The last column shows the average of Pearson's correlation coefficients between PHS and PRS.

**Table 4:** Cox regression analysis results of 5‐fold cross‐validation for PHS and PRS in GERAD dataset for individuals age at onset 55 and above

*P*‐value threshold	*N* SNPs	PHS		PRS		Pearson's correlation between PHS and PRS
		*B* [sd]	*P*‐value	*B* [sd]	*P*‐value	
5 × 10^−8^	31	0.3 [0.01]	1.3 × 10^−12^	0.29 [0.03]	5.8 × 10^−12^	0.96
10^−5^	80	0.25 [0.05]	8.4 × 10^−7^	0.31 [0.04]	5.3 × 10^−10^	0.76
10^−3^	1460	0.12 [0.04]	0.03	0.1 [0.03]	0.03	0.16
0.05	29998	0.03 [0.04]	0.42	0.004 [0.02]	0.62	0.32
0.1	49247	0.04 [0.03]	0.34	0.001 [0.02]	0.62	0.4
0.5	128952	0.05 [0.03]	0.29	0.008 [0.02]	0.59	0.59

First column shows the *P*‐value thresholds for AD associated SNP selection (from an independent IGAP_noGERAD data). Second column represents the number of SNPs that were included to the PRS/PHS score. PHS and PRS effect sizes (mean and SD) and averaged *P*‐values across 5‐fold cross‐validation subsampling are shown in columns 3–6. The last column shows the average of Pearson's correlation coefficients between PHS and PRS.

## Discussion

Polygenic risk score approach is typically used to predict risk of the disease and does not account for age at onset of cases and the fact that controls may develop the disease later in their lives. Implementation of the polygenic hazard score provides prediction for individuals’ age‐specific risk of AD development and can potentially be used for future investigation of the disease progression, intervention, and treatment of the disease.

There are two components to the risk prediction analyses by polygenic scores. The first component is the construction of the scores. Both PRS and PHS are derived as a sum of the number of risk alleles weighed by the SNP effect sizes, either logarithm of the odds ratio (log(OR)) or logarithm of the hazards ratio (log(HR)), respectively, which in turn are obtained with two different regression models (Logistic or Cox Proportional Hazard regression models). Ideally, for the construction of these scores, the SNP selection should be informed by the corresponding summary statistic from an independent dataset. For example case/control GWAS results should be used for PRS SNP selection, and Cox regression GWAS results, should be used for PHS SNP selection. The second component is the actual risk prediction. The choice of the regression model for this component of the analysis depends upon the question of interest and available data. If one is interested in predicting age‐specific risk for AD then PHS should be used as the predictor in the Cox regression model, given that the age for controls and age at onset for cases is available for the analysis.

In this study, we tested whether there is an advantage in using PHS over the PRS for age‐specific risk prediction. First, we attempted to directly replicate the results of Desikan et al.^10^ using SNPs available in the GERAD dataset. Then we constructed PRS using the same SNPs and tested how well PRS can predict the age‐specific risk in the GERAD sample. Finally, we attempted to enhance the PHS by adding more SNPs related to AD risk.

Desikan et al.^10^ selected AD associated SNPs with *P *≤ 10^−5^ in the IGAP, currently the largest publicly available GWAS dataset, then generated individual PHS in their (ADGC) dataset and tested this PHS for association with the AD age‐specific risk. We have replicated their findings in our (GERAD) data. These were as expected given previous studies, comparing LR and Cox regression,^14^, ^17^ show that the predictors with the largest effects stay significant in both Cox and Logistic regression analyses. When the effect sizes of the associated SNPs are large, the period between the age at onset and age at assessment is short (follow‐up period is 5 years or less) and survival rate is high, then both Cox regression and LR will show similar estimates of the regression coefficients.^16^ A typical AD GWAS design falls into this category. In our data the mean difference between the age of the last assessment and age at onset was 4.7 years; there were more controls than cases; and the SNPs were selected on the basis of significant association with the disease risk. Therefore, it is expected that the IGAP genome‐wide significant SNPs combined into PHS will show strong association in the ADGC and GERAD datasets as these datasets both contribute to the IGAP study.

When the SNPs were selected based upon AD association results in a sample set independent of GERAD (IGAP_noGERAD), both PHS and PRS were significantly associated with AD age‐specific risk (*P* < 10^−3^). For less stringent significance thresholds the age‐specific risk prediction was not significant with either PHS or PRS. The results of Cox regression analysis have shown no significant difference in model fits when using PRS or PHS, if SNPs are selected on the basis of strong association to AD risk. However when SNPs are prioritized for association with AD age at onset rather than general AD risk by a powerful discovery study (e.g., Cox‐regression AD GWAS), the PHS is likely to be advantageous over the PRS for age specific AD risk prediction. As such discovery study was not available to us, we could not demonstrate this advantage in our paper. We emphasize, that this is the main limitation of our study. Since we only had access to the AD GWAS summary statistics, obtained with Logistic Regression, we could not prioritize SNPs specifically associated with AD age at onset, but only used SNPs associated with AD risk overall. To overcome this issue we divided the GERAD data into a discovery and validation sub‐samples, however this reduced the power of our further analyses. Another limitation of this study is the lack of replication in a similar dataset since we did not have access to individual genotypes of another AD case/control study that was independent from IGAP. To address this limitation, we employed cross‐validation and resampling approaches to the GERAD data. The main results remained consistent in all analyses.

In conclusion, when SNPs are selected based upon case/control AD GWAS with *P*‐values thresholds up to *P *≤ 10^−3^, comparison of PRS and PHS suggests no advantage of using effects of PHS over PRS for age‐specific risk prediction. Using less stringent significance thresholds for SNP selection, the age‐specific risk prediction results are not significant by either PHS or PRS. To further enhance and validate the PHS approach for AD age at onset risk prediction, a large Cox regression GWAS needs to be conducted, and used for SNP prioritization prior to construction of the PHS for each individual.

We have demonstrated that PRS is a robust measure of the genetic liability to Alzheimer's disease. In addition to general AD risk, it predicts the age‐specific risk of AD. The PHS, when constructed using significant SNPs identified by case/control GWAS, has potential disadvantages which are similar to PRS, as outlined in Tan et al.^18^ Development of PHS based up on Cox Hazard Regression model GWAS is a potential way forward to validate the PHS approach.

## Author Contribution

GL, RS, and MS involved in data analysis, drafting the manuscript, or part of it. AF involved in data analysis. PB, GS, and NF involved in critical review of the manuscript. JW, JH, and VE‐P involved in conception and design of the study, drafting and critical review of the manuscript.

## Conflicts of Interest

JH‐grants from Cytox, outside the submitted work; JW‐patent for diagnostics on some SNPs identified as associated with AD; VE‐P – personal fees from Consultancy, outside the submitted work. GL, RS, MS, AF, PB, GS, and NF have nothing to report.

## Supporting information


**Table S1**. Sample size and descriptive statistics for the GERAD dataset. Legend.

**File S1.** GERAD authorship list.
