# Supplementary figures and images for: Polygenic risk and hazard scores for Alzheimer's disease prediction
Source: Ann Clin Transl Neurol. 2019 Feb 18;6(3):456–65. doi: 10.1002/acn3.716 (PMC6414493; doi:10.1002/acn3.716)

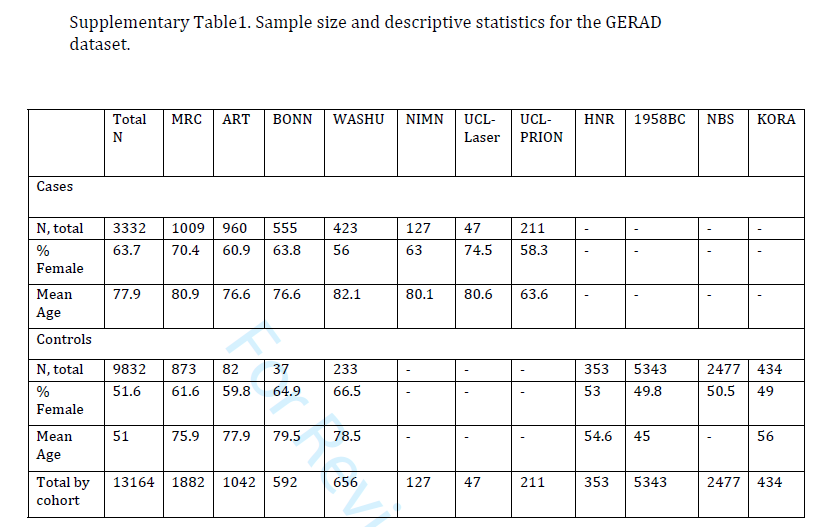

Supplement: Supplementary file 1 — Table S1. Sample size and descriptive statistics for the GERAD dataset. Legend. [file ACN3-6-456-s001.docx]
